# ^29^Si solid state NMR and Ti *K*-edge XAFS pre-edge spectroscopy reveal complex behavior of Ti in silicate melts

**DOI:** 10.1186/s40645-020-00326-2

**Published:** 2020-03-18

**Authors:** Michael R. Ackerson, George D. Cody, Bjorn O. Mysen

**Affiliations:** 1 Department of Mineral Sciences, Smithsonian National Museum of Natural History, PO Box 37012, Washington DC, 20013-7012 USA; 2 Geophysical Laboratory, Carnegie Institution of Washington, 5251 Broad Branch Road NW, Washington DC, 20015 USA

## Abstract

An understanding of the mechanisms of Ti is incorporation into silicate glasses and melts is critical for the field of petrology. Trace-element thermobarometry, high-field-strength element partitioning, and the physical properties of magmas are all be influenced by Ti incorporation into glasses and changes therein in response to changes in composition and temperature. In this study, we combine ^29^Si solid state NMR and Ti *K*-edge XAFS spectroscopy to investigate how Ti is incorporated into quenched Na-silicate glasses, and the influence of Ti on the structure of silicate species in these glasses. ^29^Si NMR shows that in both Ti-bearing Na_2_O•4SiO_2_ (NS4) and Na_2_O•8SiO_2_ (NS8) glasses, increasing the amount of Ti in the melt results in a shift of Si Q^4^ peak in the ^29^Si NMR spectra reflecting Ti nearest neighbors for Si in Q^4^ speciation. The Ti XAFS results from NS8 glass indicate that Ti is primarily incorporated in [5]-fold coordination. At higher Ti content, there is a shift of the XAFS pre-edge feature suggesting mixing of [4]-fold Ti into the spectra. Combined, the ^29^Si NMR and XAFS pre-edge data are consistent with Ti incorporation as isolated ^[5]^Ti atoms and the formation of ^[5]^Ti clusters at relatively low Ti concentrations, with no evidence for Ti–Na interactions as suggested by previous studies. As the Ti content increases, the Ti atoms begin to occupy 4-fold coordinated sites interacting primarily with Si in Q^4^ speciation (no significant Na–^[4]^ Ti bonding). The internal consistency of these two techniques provides a uniquely complete snapshot of the complexity of Ti incorporation in silicate melts and underlies the importance of understanding Ti incorporation mechanisms in natural magmatic systems.

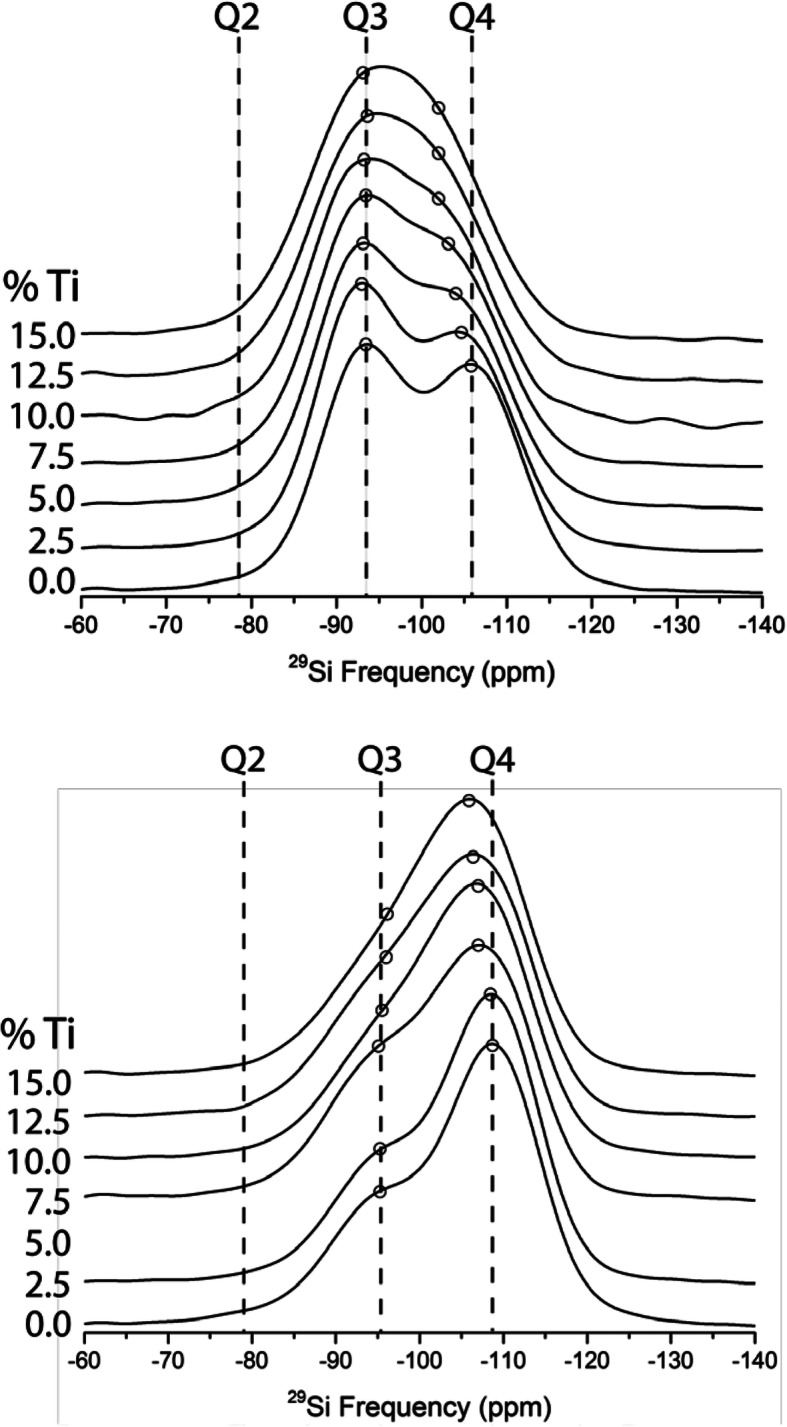

## Introduction

Titanium, typically a minor element in natural magmatic systems, does nevertheless, play a major role in the evolution of igneous and metamorphic rocks. Ti-rich oxide minerals control trace-element budgets in magmas and metamorphic melting reactions (Ryerson and Watson 1987; Tang et al. 2019; Xiong 2006), Ti activity in magmas is a critical component of trace-element thermobarometry (Ackerson et al. 2018; Ghiorso and Gualda 2013; Watson and Harrison 2005), and Ti incorporation imparts unique structural and physical properties (e.g., refractive index, tensile strength, compressibility) into melts and glasses (Liska et al. 1996; Liu and Lange 2001; Morsi and El-Shennawi 1984; Roskosz et al. 2004; Scannell et al. 2015). All these characteristics are controlled by the mechanisms of Ti incorporation (solubility) into melts/glasses, yet our understanding of these solubility mechanisms is fundamentally limited.

The Ti solubility in magmatic liquids varies over many orders of magnitude, from 1000 s of ppm in silicic magmas (Hayden and Manning 2011; Hayden and Watson 2007) to over 20 wt% in intermediate and mafic glasses (Gaetani et al. 2008). This behavior is likely due to the number of potential solubility mechanisms for Ti in magmas. In various natural and synthetic glasses and using multiple analytical techniques [e.g., Raman (Henderson and Fleet 1995; Mysen and Richet 2005), XAFS (Farges 1997; Farges et al. 1996a), molar volume calculations (Lange and Navrotsky 1993)], Ti has been demonstrated to be incorporated in [4]-, [5]-, and [6]-fold coordination. In [4]-fold coordination Ti acts primarily as a network-forming cation, helping to polymerize the melt, whereas [6]-fold coordinated Ti will act as a network-modifying cation (Mysen and Richet 2005, references therein). Ti also has the potential to cluster in melts, particularly in [5]- and [6]-fold coordination, where ^[5]^Ti is suggested to form Na-titanite isolates and clusters (Farges et al. 1996a; Yarker et al. 1986).

One potential way to investigate Ti solubility and its influence on melt structure is to observe the effect of Ti on other cations in quenched glasses. ^29^Si NMR provides the opportunity to do this, as it can be used to resolve how addition of Ti to magma will affect the degree of polymerization and the bonding environment of Si. A combination of ^29^Si NMR and Ti *K*-edge XAFS pre-edge spectroscopy (which provides information on the average coordination of Ti in glasses) can give a more complete picture of how Ti is incorporated into silicate glasses. In this study, we utilized these two techniques on a series of synthetic Ti-bearing Na-silicate glasses to observe how Ti is incorporated into glasses formed by temperature quenching of melt and how Ti solubility mechanisms may be affected by Ti concentration.

## Experimental and methods

### Titano-sodium silicate synthesis

**Fig. 1:**
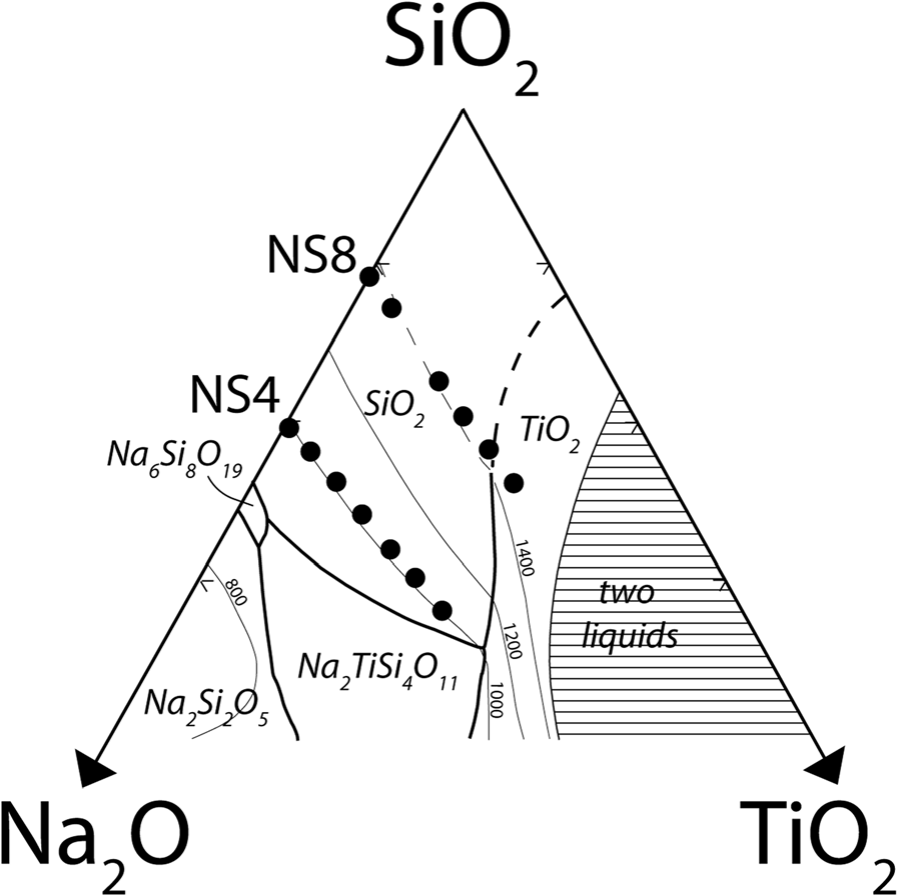
Upper portion of the SiO_2_–Na_2_O–TiO_2_ phase diagram. Our experiments were performed along the NS8 and NS4 joins. Experimental bulk compositions are denoted with black circles. Thin black lines are liquidus isotherms, and thick black lines are liquidus phase boundaries. (modified from Glasser and Maar 1979)

In this study we have focused on investigating the perturbation in silicate melt structure (as observed in quenched glasses) through the addition of TiO_2_ along a simple composition join linking a binary sodium silicate composition with TiO_2_ (Fig 1). We focus on two specific compositions, those of Na_2_O•8SiO_2_ (NS8) and Na_2_O•4SiO_2_ (NS4), with increasing mol% addition of TiO_2_ from 2.5 up to 15 mol% (Fig. 1). Note that at concentrations above 15 mol%, we are close to the liquid-liquid miscibility gap (Glasser and Maar 1979). Glasses were synthesized from SiO_2_, TiO_2_, and Na_2_CO_3_ starting material by heating at a rate of 100 °C/h from 700–900 °C, then at a rate of 150 °C/h to 1400 °C in Pt crucibles. Melts were held at 1400 °C for 1 h, after which they were quenched to room temperature. In air, the glasses quenched to below the glass transition temperature in less than 30 s, owing to the small size of the Pt crucibles used. The glass structure recorded in the glasses is that of the glass at the glass transition temperature. For this study, and because of the potential for temperature dependence on the solubility mechanisms (Lange and Navrotsky 1993), all glasses were synthesized at 1400 °C for 1 h. Several NS8 glasses with 10 mol% TiO_2_ were also synthesized at multiple temperatures to test for a potential temperature dependence on XAFS spectra (supplementary fig. S2). The glasses were inspected in immersion oil under a petrographic microscope to ensure no crystalline phases were present, and to ensure there were no signs (e.g., cloudiness, opalescence) indicating the presence of nanocrystalline anatase (Henderson and Fleet 1995). Furthermore, no anatase was detected in these glasses in X-ray diffraction (XRD) spectra (Ackerson and Mysen in press).

### ^29^Si solid state nuclear magnetic resonance spectroscopy

All solid state ^29^Si NMR spectra were acquired using a Chemagnetics Infinity solid state NMR spectrometer that employs a 7 Tesla static magnetic field. The resonant frequency of ^29^Si at ~ 7 Tesla is 59.6 MHz. Crushed glass samples were loaded into a 5 mm diameter rotor and placed within a Chemagnetics double resonance magic angle spinning (MAS) probe. Following Maekawa et al. 1991, the addition of a paramagnetic relaxation agent (Fe_2_O_3_ at 0.1 wt%) results in a significant reduction in ^29^Si’s spin lattice relaxation (T_1_) time without spectral distortion. Tests on NS4 glass revealed that a pulse width of 1.3 μs, corresponding to a 30° nutation angle and recycle delays spanning from 2 to 20 s did not reveal any reduction in intensity resulting T_1_ saturation; hence, a recycle delay of 2 s was chosen for all compositions. The MAS frequency (ω_r_/2π) was 8 KHz. The number of acquisitions was in each case 40 K and the ^29^Si spectra were referenced to the ^29^Si frequency of tetramethylsilane (TMS) defined as 0 ppm.

### Ti *K*-edge pre-edge X-ray absorption fine structure (XAFS)

XAFS spectra were collected on a suite of Ti-bearing NS8 glasses at beamline 13 IDE at the Advanced Photon Source at Argonne National Laboratory. The beam was filtered using the Si (111) monochromator, tuned to the absorption edge at 4964.46 eV, and was calibrated on Ti foil. Relative to the absorption edge, scans were conducted over a dynamic range with an emphasis on collecting high energy resolution spectra in the pre-edge region: from − 60 to − 6 eV data were collected at a 2 eV step for 2 s, from − 6 to 15 eV at a 0.1 eV step for 2 s, and a 2 eV step for 2 s from 15 to 200 eV. Data were processed using the Larch software (Newville 2013). Each spectrum was processed by first defining the absorption edge at the maximum first derivative of the spectra within the edge step region (care was taken not to inadvertently select the maximum first derivative of the pre-edge feature), then applying a linear normalization to the pre-edge region (set to 0) and edge step (set to 1), focusing on normalization of the XAFS region (see Ackerson et al. 2017 for normalization details).

Pre-edge peaks were fit with pseudo-Voigt peaks to determine the precise position of the peak centers (Farges et al. 2004). Linear mixing was performed for three end-member pre-edge spectra to determine the position of the pre-edge peak as a function of mixing coordination states. End-member Ti-bearing forsterite ([4]-fold), fresnoite ([5]-fold), and titanite ([6]-fold) were used as end-members for mixing curves. Peak heights of the primary peak were used to determine mixed peak positions in lieu of peak centroids since [6]-fold coordinated Ti end-member spectra have multiple discrete pre-edge peaks. Importantly, the influence of these complex features on spectra diminishes rapidly during mixing, so their net influence on the spectra is less than the precision of the mixing technique (i.e., the ~+/− 10% mixing estimates for coordination mixing is greater than the influence of complex [6]-fold peaks).

## Results and discussion

The NS4 and NS8 compositions utilized in this study have been extensively studied for the purpose of elucidating the influence of volatiles and network-forming and network-modifying cations on melt structure (Cody et al. 2005; Kuemmerlen et al. 1992; Maekawa et al. 1991; Mysen et al. 2011; Roskosz et al. 2006; Zotov and Keppler 1998), and were chosen specifically as they provide a model system for studying moderately polymerized and chemically evolved magmatic liquids. Additionally, sodium silicate compositions are favored for structure vs. composition studies is because the key structural elements of silicate glasses and melts (the so called “Q^n^” species (Lippmaa et al. 2002), see discussion below) are clearly resolved using ^29^Si solid state NMR.

Sodium is a network modifying cation, which means that addition of Na_2_O to a silicate structure will yield non-bridging oxygens (NBOs). The structure of silicate melts and glasses quenched from them is characterized, in part, by the number of bridging oxygens per silicate oxide tetrahedron. These are often described as Q^n^-species, which are defined by the number of bridging oxygens per tetrahedrally coordinated cations of the silicate structure (*n* = no. of bridging oxygens, e.g., Q^4^-Q^0^). In the case of the sodium silicate compositions explored here, given the relatively high silica content, the ^29^Si solid state NMR reveals only Q^3^ and Q^4^ species well resolved from each other (Cody et al. 2005; Kuemmerlen et al. 1992; Maekawa et al. 1991; Zotov and Keppler 1998).

### Silicon (^29^Si) solid state NMR data

^29^Si is the only silicon isotope (of three: ^28^Si, ^29^Si, and ^30^Si) with spin (*S* = 1/2 in this case), with a natural abundance of 4.7%. In the present case, all silicon is in the 4^+^ valence state and tetrahedrally bonded to four oxygen atoms.

**Fig. 2:**
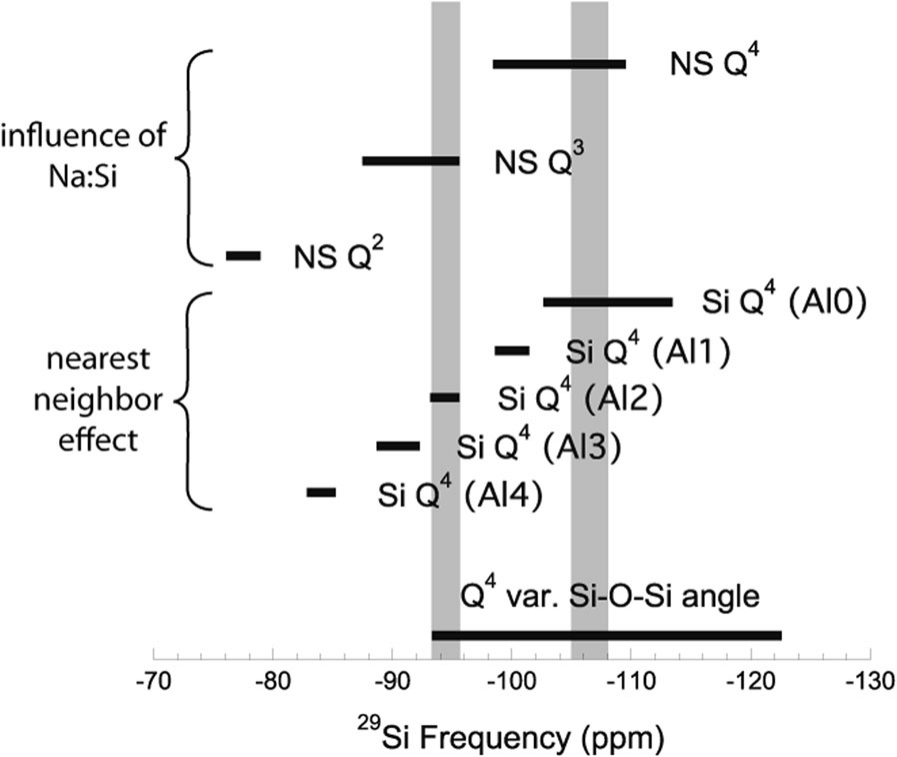
Factors influencing peak positions of Q-species in 29Si NMR spectra. Grey vertical bars are the peak frequencies observed in the present study. The number of non-bridging oxygens (number of Si–O–Na interactions) causes a positive shift in peak frequency, such that Si in Q^2^ speciation (2 non-bridging oxygens) is at a higher frequency than Si in Q^4^ speciation (0 non-bridging oxygens). For Si in Q^4^ speciation, Al as a network-forming cation nearest neighbor has been shown to cause a shift toward higher frequency. Q^4^ Si with one Al nearest neighbor (Al1) is at a lower frequency than Q^4^ Si with 4 Al nearest neighbors (Al4). Variations on Si–O–Si bond angle can also induce large shifts in the Q^4^ peak position, shown here from lower frequency (− 122 ppm at 170°) to higher frequency (− 93 ppm at 130°). These variations in bond angle are the cause of the Al nearest neighbor effects (Lippmaa et al. 1981)

In simple silicate systems such as NS8-TiO_2_ and NS4-TiO_2_, there are three primary factors that affect the ^29^Si solid state NMR spectrum. First, there is the approximately + 10 ppm shift accompanying changes in NBO (i.e., moving from Q^4^ to Q^3^ to Q^2^ … etc.) (Maekawa et al. 1991; Magi et al. 1984) (Fig. 2). As noted by Maekawa et al. 1991, there is a systematic shift in Q^4^ and Q^3^ frequency with increasing sodium content, where the range in shift is greatest for Q^4^ and decreases with increased NBOs. For any given NS composition, the variation in shift (evident in bandwidth) is much less than indicated in Fig. 2: whereas the bands at the top of the figure show bandwidth for a wide range of NS compositions, the positions of Q^4^ and Q^3^ for NS8 and NS4 much narrower (shown with gray regions in Fig. 2).

A second factor that governs ^29^Si shift (in the present case) is the perturbation to the ^29^Si frequency related to substitution of next nearest network cations with elements other than silicon (e.g., with aluminum) as has been studied by Lippmaa et al. 1981 (Fig. 2). In this case, the effect on ^29^Si frequency in a Q^4^ species with sequential substitution Al^3+^ for Si^4+^ induces a ~+ 5 ppm shift per substitution (Fig. 2) (Lippmaa et al. 1981). The physics underlying this shift is understood to involve differences in the electronegativity of Al^3+^ relative to Si^4+^ (Janes and Oldfield 1985). While this effect is well documented for aluminum substitution, as far as we can find, no such information is available for the perturbation resulting from titanium (Ti^4+^) substitution for Si^4+^. We do note, however, that the electronegativity of Ti^4+^ is much closer to that of Al^3+^ (Pauling 2014) than Si^4+^, we, therefore, expect the perturbation on the ^29^Si NMR signal due to titanium nearest neighbors to be similar to that of aluminum substitution.

The third major perturbation on ^29^Si NMR frequencies is any variation in Si–O–Si bond angle. As shown in Fig. 2 for ^29^Si in Q^4^ a variation in Si–O–Si angle from 170 to 130° results in a range in frequency from − 122 ppm up to a frequency of − 93 (Mauri et al. 2000; Smith and Blackwell 1983). This is just for one Q species. If multiple Q species are present with such variation, the ^29^Si NMR spectrum becomes extremely broad and detailed structural interpretation is very difficult. It is notable that wide variation in NS Q^4^ (Fig. 2) ^29^Si NMR frequency as well as that observed for Si Q^4^ (Al0) is most likely due to variation in Si–O–Si bond angle. For example, (Le Losq et al. 2015) showed that lithium, sodium, and potassium tetrasilicate glasses, there is a systematic shift in Si–O–Si bond angles in Q4 species, where from Li^+^ to K^+^ the bond angles decreased.

**Fig. 3:**
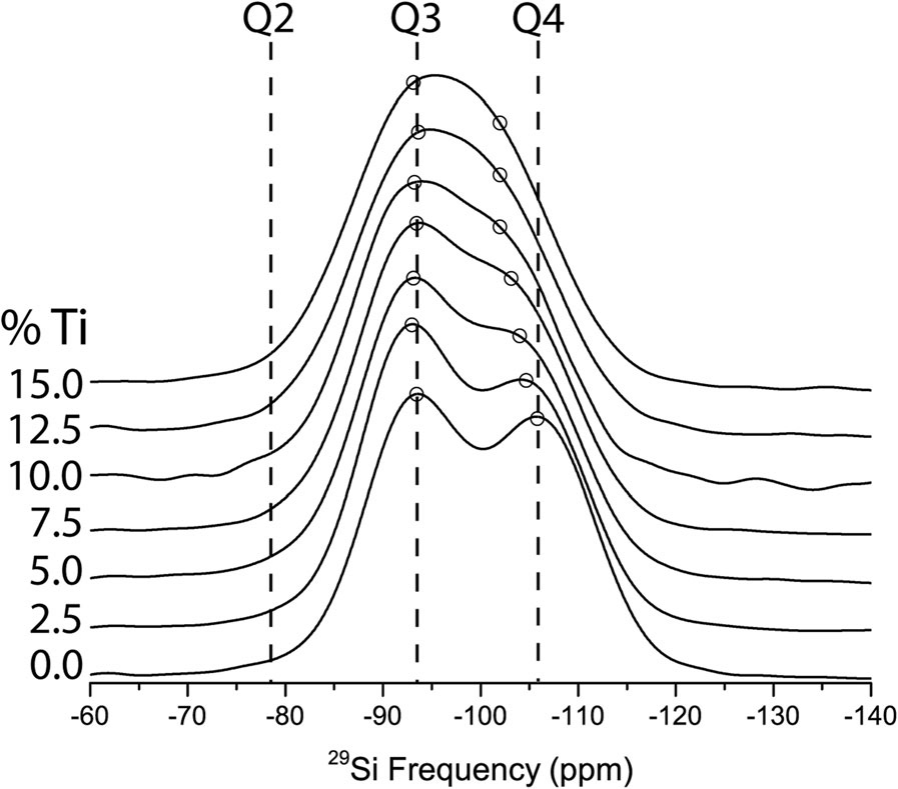
Stacked ^29^Si NMR data for NS4 glass at various Ti concentrations, normalized by integrated intensity. The two predominant peaks are the Q^3^ and Q^4^ peaks at − 93 and − 106 ppm, respectively. At 0% TiO_2_, the two peaks account for nearly equal areas under the curve, as is expected for NS4 glasses with 50% Q^3^ Si and 50% Q^4^ Si. There is a shift in peak position of the Q^4^ to higher frequency with addition of Ti to the NS4 glass, whereas the Q^3^ peak is negligibly affected by the addition of Ti. The shift in Q^4^ to higher frequency is likely the result of Ti nearest neighbor effects on the Q^4^ Si. Open circles are fitted peak centers

**Fig. 4:**
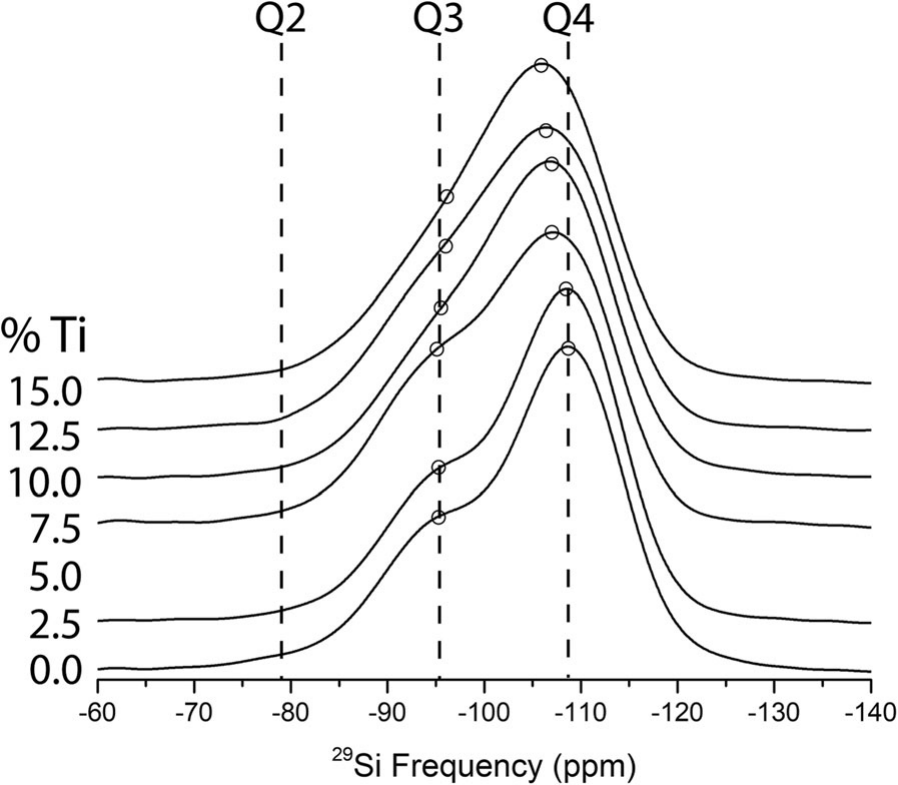
Stacked ^29^Si NMR data for NS8 glass at various Ti concentrations, normalized by integrated intensity. Peaks at − 96 and − 109 ppm are Q^3^ and Q^4^, respectively. Compared with the NS4 glasses, Ti-free NS8 glass exhibits a more pronounced Q^4^ peak, as is predicted based on the Na:Si of the glass. As with the NS4 glasses, there is a progressive positive shift in peak energy of the Si Q^4^ peak, likely as a result of Ti nearest neighbors for the Si Q^4^ species. Open circles are fitted peak centers

**Fig. 5:**
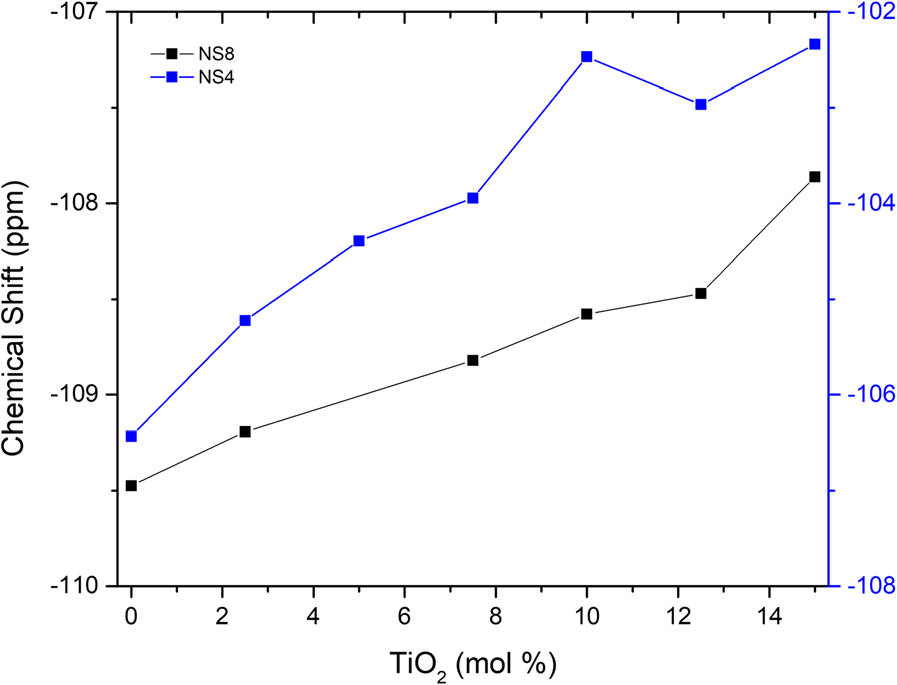
Position of the ^29^Si NMR Q4 peak as a function of bulk TiO_2_ content of the glasses for NS4 (blue) and NS8 (black) compositions. The systematic shift in frequency is likely due to the continuous growth of an additional Q4 peak due to ^[4]^Ti nearest-neighbor interactions with Q4 Si. Peak fitting of the spectra was performed using Gaussian peaks, with fit iterations converging at a chi-squared tolerance value of 1E-6

Figures 3 and 4 present ^29^Si solid state NMR spectra of the TiO_2_ containing NS glasses (NS8 and NS4). In the case of the NS4 glass (Fig. 3), one observes (at 0% TiO_2_), two peaks corresponding to Q^4^ and Q^3^ species, at − 105.5 and − 93.3 ppm, respectively. Note that although the Q^3^ peak appears more pronounced than the Q^4^ peak, the Q^4^ peak is slightly broader and the peak areas for NS4 are nearly identical as would be predicted based on stoichiometry. With increasing TiO_2_, there is an immediate and obvious shift of the Q^4^ peak to slightly higher frequency, while no such apparent shift in the Q^3^ peak is evident. In the case of the NS8 glass (Fig. 4), with increasing TiO_2_, there is also shift in the Q^4^ peak to slightly higher frequency, but the magnitude of the shift with increasing TiO_2_ is less than that of the NS4 + TiO_2_ glasses. As with the NS4 glasses, there is no obvious change in frequency or relative intensity of the Q^3^ species in NS8 + TiO_2_ glasses. The shift of the Q^4^ peak position in NS4 and NS8 glasses to progressively higher frequencies with increasing TiO_2_ is shown in Fig. 5.

### Ti XAFS Spectroscopy

Ti *K*-edge XAFS pre-edge spectra can provide information on the average coordination of Ti in minerals and glasses. The pre-ionization edge feature of the Ti *K*-edge XAFS spectra is produced by 1 s-3d orbital transitions, where the intensity of the peak is positively correlated with the degree of d-p orbital mixing (which will be greater with decreasing coordination), and the peak energy is negatively correlated with d-p mixing (Farges et al. 1996b; Waychunas 1987). The peak intensity and energy of the pre-edge peaks for the three coordination states for Ti in glasses ([4]-, [5]-, and [6]-fold coordination) are, therefore, resolvable, making these energies useful for estimating the average Ti coordination in the quenched glasses considered here.

**Fig. 6:**
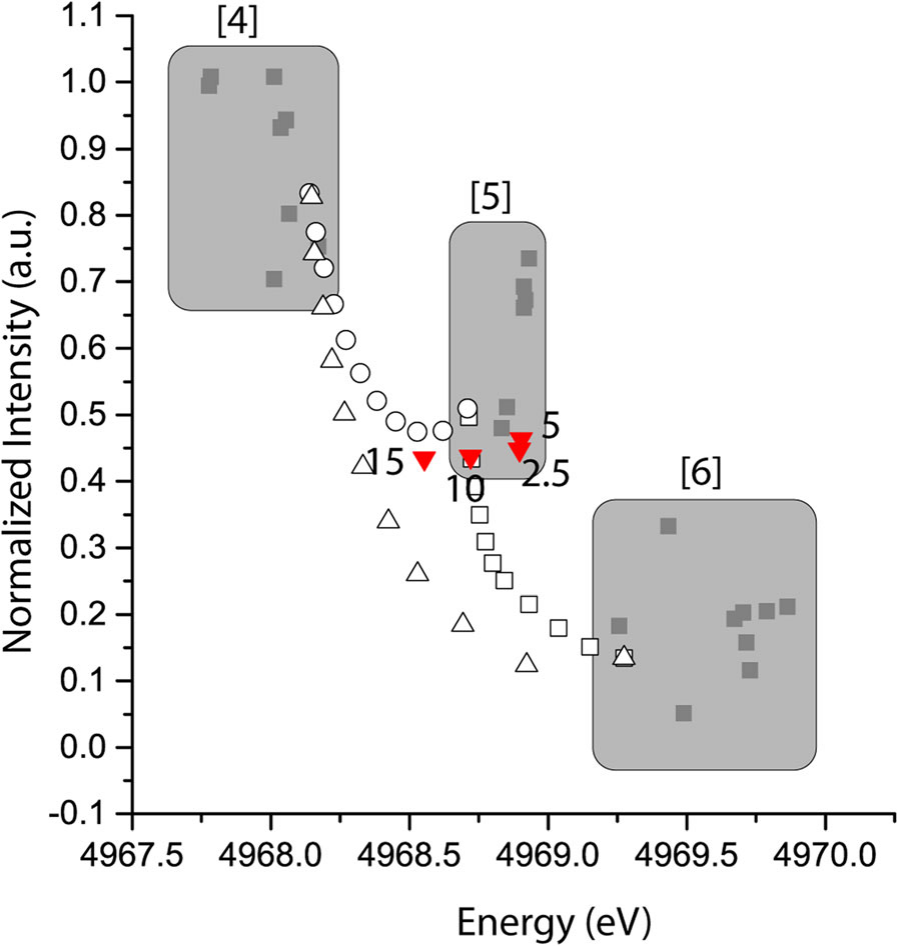
Ti k-edge X-ray absorption fine structure (XAFS) pre-edge peak positions and mixing curves. The Ti pre-edge feature is sensitive to the coordination environment and is used to assess average Ti^4+^ coordination in the experimental glasses. Grey regions and grey, filled symbols are the pre-edge peak positions of minerals and glasses with known Ti coordination (Farges et al. 1996b). White filled symbols are mixing curves between end-member ^[4]^Ti-bearing forsterite, ^[5]^Ti-bearing fresnoite, and ^[6]^Ti-bearing titanite. Red symbols are the peak positions of NS8 glasses with the mol% TiO_2_ indicated next to the symbols. As Ti concentration increases, the peak positions shift along a trend similar to the mixing of ^[4]^Ti and ^[5]^Ti, indicating the average coordination of Ti in the glasses decreases with increasing bulk TiO_2_ content

In previous studies of Ti-bearing glasses, pre-edge features of Ti-bearing glasses have shown that Ti exists in multiple coordination states ranging from nearly complete octahedral to complete tetrahedral coordination (Farges et al. 1996b; Romano et al. 2000). Mixed Ti coordination results in pre-edge features that are linear combination of the end-member spectra, a phenomena which can be utilized to estimate the amount of Ti in multiple coordination states (Farges 1997). In the present study, we employed XAFS to analyze selected glasses from the NS8 series and several mineral standards of known Ti coordination to verify the nature of Ti coordination and explore the potential for coordination mixing in the glasses as a function of bulk TiO_2_ content (Fig. 6).

For a given end-member coordination state, there is significant variability in the pre-edge peak intensity and energy, which limits the precision of determining the degree of coordination mixing (Farges 1997; Farges et al. 1996b) for systems like the NS glasses studied here whose end-member Ti coordination peak positions and energies are unknown. In lieu of known end-member composition for Na-silicate glasses for any coordination state, we utilize forsterite, fresnoite, and titanite as Ti^4+^ [4]-, [5]-, and [6]-fold coordination standards, respectively.

For the NS8 glasses analyzed, there is a subtle but significant trend in the pre-edge peak shifting toward lower energy and slightly lower intensity with an increase in bulk TiO_2_. At low Ti content, the peak position is closest to end-member fresnoite, indicating that almost all Ti occurs in [5]-fold coordination. As Ti content increases, the peak systematically shifts toward lower energy by ~ 0.35 eV. As seen in the end-member mixing curves (Fig. 6), this trend is similar (albeit shifted) to the trend in mixing ~ 40% ^[4]^Ti to end-member ^[5]^Ti, which from the known compound model mixing will produce a ~ 0.33 eV shift. There is no indication from these results that significant ^[6]^ Ti exists in the NS8 glasses. XANES spectra also confirm that the material is glassy, with no micro- or nano-scale crystallization (supplementary figure S1).

Results from Ti pre-edge XAFS and ^29^Si NMR provide three critical observations that aid in interpreting structural changes in the glasses with changes in bulk TiO_2_ content:

1. At low TiO_2_ content, Ti is primarily in [5]-fold coordination. With increasing bulk TiO_2,_ the amount of [4]-fold coordinated Ti increases;
2. With increasing Ti, there is no apparent growth of the ^29^Si Q^4^ peak, but rather an apparent shift toward higher energy of the Q^4^ peak;
3. There is no frequency shift, peak growth, or apparent peak diminution of the ^29^Si Q^3^ with variations in TiO_2_ content.

Previous research into Ti-bearing NS2 and NS4 glasses suggests that ^[5]^Ti is incorporated as Na-titanite complexes (Farges et al. 1996a). Na-titanite formation in NS4 and NS8 glasses would consume Na^+^ and result in an observed polymerization of Si via the growth of the ^29^Si Q^4^ peak at the expense of Q^3^ Si in the NMR spectra, which is not observed in either the NS4 or NS8 glasses. In a Ti-free NS8 glass, 25% of Si atoms are bonded to non-bridging oxygens through Si–O–Na interactions. Removal of Na^+^ from these bonds via Na-titanite complexes would force the polymerization of Si. In the Si^29^ NMR spectra, this would be seen in the growth of the Q^4^ peak at the expense of the Q^3^ peak. For example, if each ^[5]^Ti atom scavenged three Na atoms (Farges and Brown 1997), at 6.25 mol% TiO_2_, Si^4+^ in NS8 glass would be completely in Q^4^. In our system, there is no indication that the ^29^Si Q^4^ peak grows at the expense of the Q^3^ peak, requiring that Na–Ti bonding is minimal to absent along the NS4 and NS8 TiO_2_ glasses. It is noted that the previous research that indicated the presence of Na-titanite complexes (Farges et al. 1996a) was performed on more Na-rich (e.g., NS2) glasses. These glasses are in Na-silicate and Na-Ti-silicate liquidus fields (Fig. 1), as opposed to the SiO_2_ and TiO_2_ liquidus fields of NS4 and NS8 glasses. In these systems where Na activity is higher, it would be expected that Na-titanite complexes could form in the melt as suggested in Fig. 1.

In the case of NS4 and NS8 titanium bearing glasses, the presence of five coordinated Ti could be incorporated via a charge-balancing mechanism that does not rely on Na-titanate clusters. For example, titanium complexes with both 3 and 2 coordinated oxygens, ^[5]^T^i4+^ + 3^[3]^O^−3/2^ + 2^[2]^O^−1^. In this case, Ti would form ^[5]^Ti clusters at low concentrations through sharing of ^[3]^O between 3 ^[5]^Ti atoms, and ^[2]^O could interact with ^[5]^Ti, ^[4]^Si, or ^[4]^Ti. This mechanism helps to explain both the apparent shift in Q^4^ (as opposed to diminution of Q^3^) peaks for the ^29^Si observed in the NMR spectra as well as the initial pure ^[5]^Ti observed in the Ti XAFS pre-edge region. This type of ^[5]^Ti bonding accounts for both the XAFS and NMR observations, and suggests that even at low bulk Ti content, ^[5]^Ti is bonded primarily through clustering mechanisms with negligible Na–Ti interactions.

Because TiO_2_ is being added in excess to this system (as opposed to being added as an equimolar replacement of SiO_2_), we envision Ti exerting an effect similar to oxygen triclusters that have systematically been observed in peraluminous melts, where the amount of Al in the system is in excess of charge-balancing alkali cations (Stebbins et al. 2001; Toplis et al. 1997). Alternatively, if the influence of Ti on Si NMR is similar to that of Al in these glasses, it is possible that there is a diminution of the Q^3^ peak as a result of a concomitant growth of a Q^4^ (Ti = 2) peak. Although we do not this this is the most likely scenario given the data collected, it is difficult to specifically discount this mechanism given the broadness of the peaks at increasing bulk TiO_2_ content.

**Fig. 7:**
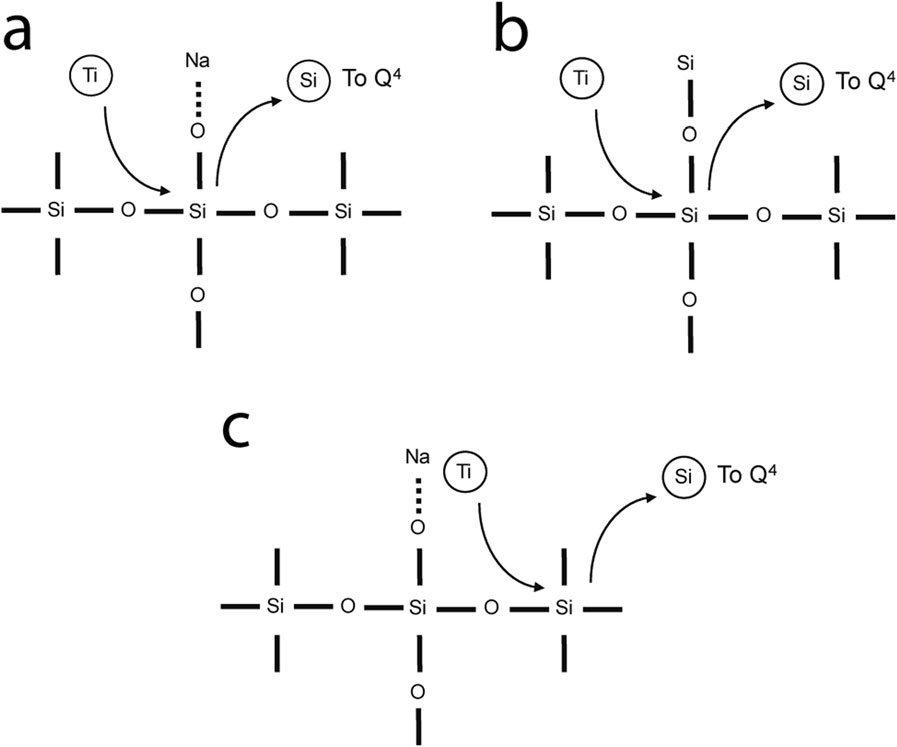
Three different mechanisms by which ^[4]^Ti can be incorporated into the glass structure include: **a** in replacement of Q^3^ Si, which would induce a loss of Q^3^ Si and growth of Q^4^ Si in the NMR spectra, which is not observed, **b** for Q4 Si in the network with no adjacent Q3 Si, which would incite the growth of an additional Q4 peak at a slightly higher energy than the primary Q4 peak, and **c** for Q4 Si adjacent to Q3 Si, which would incite a peak shift toward higher frequency of the Q3 Si peak. The latter is not observed in the NS8 glasses, but a slight shift in peak position is detectable in the NS4 glasses (Fig. 3)

The ^29^Si NMR data indicate that as Ti concentration increases, incorporation of ^[4]^Ti occurs predominantly through the interaction of Ti with Q^4^ Si, as a network-forming cation in Q^4^ speciation or as will be shown later as isolated Ti clusters. In Fig. 7, three ^[4]^Ti^4+^ substitution schemes are considered. Naturally, this figure is not meant to represent all Ti in the system (most Ti is in five-fold coordination), but rather to address potential substitution mechanisms for ^[4]^Ti^4+^. In Fig. 7a, Ti^4+^ substitution for Si^4+^ in a Q^3^ species with the neighboring sodium cation providing charge balance is envisioned. From the perspective of the ^29^Si NMR spectra, where this substitution to be significant, one would observe a reduction in Q^3^ intensity and an increase in Q^4^ intensity, where such spectral changes are not observed in either Fig. 3 or 4.

In the scenario where Ti^4+^ substitutes for Si^4+^ in Q^4^ species (Fig. 7b), from a perspective of ^29^Si NMR, one would expect to see a shift in Q^4^ to a higher frequency if the effect of titanium coordination is similar to the case of Al^3+^ substitution into Si^4+^ Q^4^ species (Fig. 2). This shift is the result of the growth of a new Q^4^ peak at the expense of the original Q^4^ peak, we expect this new Q^4^ (Ti = 1) peak to occur at a slightly higher frequency of the Q^4^ (Ti = 0) peak similar to the effect of Al substitution (Fig. 2). In both NS4 and NS8 (with TiO_2_), the ^29^Si NMR frequency shift of the “Q^4^” peak supports the scenario that ^[4]^Ti^4+^ substitutes for Si^4+^ in Q^4^ species. Note that in Fig. 7c, a Ti^4+^ substitution into a Q^4^ species adjacent to a Q^3^ species would also be expected to impart a small positive frequency shift in ^29^Si Q^3^; however, no such shift (as is observed with Q^4^) is evident for the NS8 + TiO_2_ glasses, but in the case of NS4 + TiO_2,_ there is possibly evidence of the growth of very weak signal at frequencies at and above − 80 ppm, potentially suggesting the growth of a broad and very weak peak buried below the prominent Q^3^ peak as will be considered below.

For the purpose of completeness, we note that it is conceivable that a mechanism might exist wherein titanium solution into silicate rich (Q^4^) domains draws silica away from Q^3^ domains, which would necessitate the formation of Q^2^ species. In the present case, there is no ^29^Si NMR evidence for any development of Q^2^ (Fig. 2) and, therefore, there is no evidence to support this potential scenario for these melt (glass) compositions. It is noted that in a Raman study along the NS2-NT2 join (Mysen and Neuville 1995) that the silicate NBO increased as NT2 increased implying behavior that is consistent what is observed for the NS4-TiO_2_ and NS8-TiO_2_ glasses studied here.

Based on the ^29^Si NMR data presented in Figs. 3 and 4, it therefore appears that the most conservative interpretation is that for high silica content sodium silicate melts (e.g., NS4 and NS8 analyzed as glasses), addition of ^[4]^TiO_2_ predominantly perturbs the silicate rich (Q^4^) domains and minimally (if at all) perturbs the sodic rich domains (Q^3^). Given that NS8 has 3 times more Q^4^ relative to Q^3^ than NS4, it is perhaps not surprising that the magnitude of the perturbation to ^29^Si NMR is less in NS8 glasses with equivalent increases in TiO_2_ (see Figs. 3 and 4).

The ^29^Si NMR spectra in Figs. 3 and 4 are quite broad at higher Ti compositions and there are little to no constraints to perform robust spectral fitting—this is particularly the case for NS4-Ti (Fig. 3). Therefore, instead of fitting the spectral data, we use the known effect of Al^3+^ substitution on Si Q^4^ frequency as a guide (Fig. 2) (Lippmaa et al. 1981) for what Ti^4+^ would be expected to do, i.e., cause the formation of a new peak at ~ 100 ppm due to Q^4^ (Ti = 1) species. We then explore how the ^29^Si spectrum could be progressively perturbed with increased titanium addition by forward modeling the growth of this new Q4 (Ti = 1) peak with proportional loss of Q4 (Ti = 0) peak intensity. We test the validity of the forward models by whether these model spectra replicate what is observed in the actual data. As noted previously and shown in Fig. 2, the ^29^Si peak for Si Q^4^ with one aluminum nearest neighbor (Q^4^ Al = 1) lies between NS4 and NS8 Q^3^ and Q^4^ (at 0% TiO_2_) for both NS4 and NS8. For modeling purposes, we assume that the peak for Q^4^ (Ti = 1) also lies at between Q^3^ (Ti = 0) and Q^4^ (Ti = 0) based on the idea that the similarity of electronegativity with Ti^4+^ and Al^3+^ will result in a similar chemical shift.

**Fig. 8:**
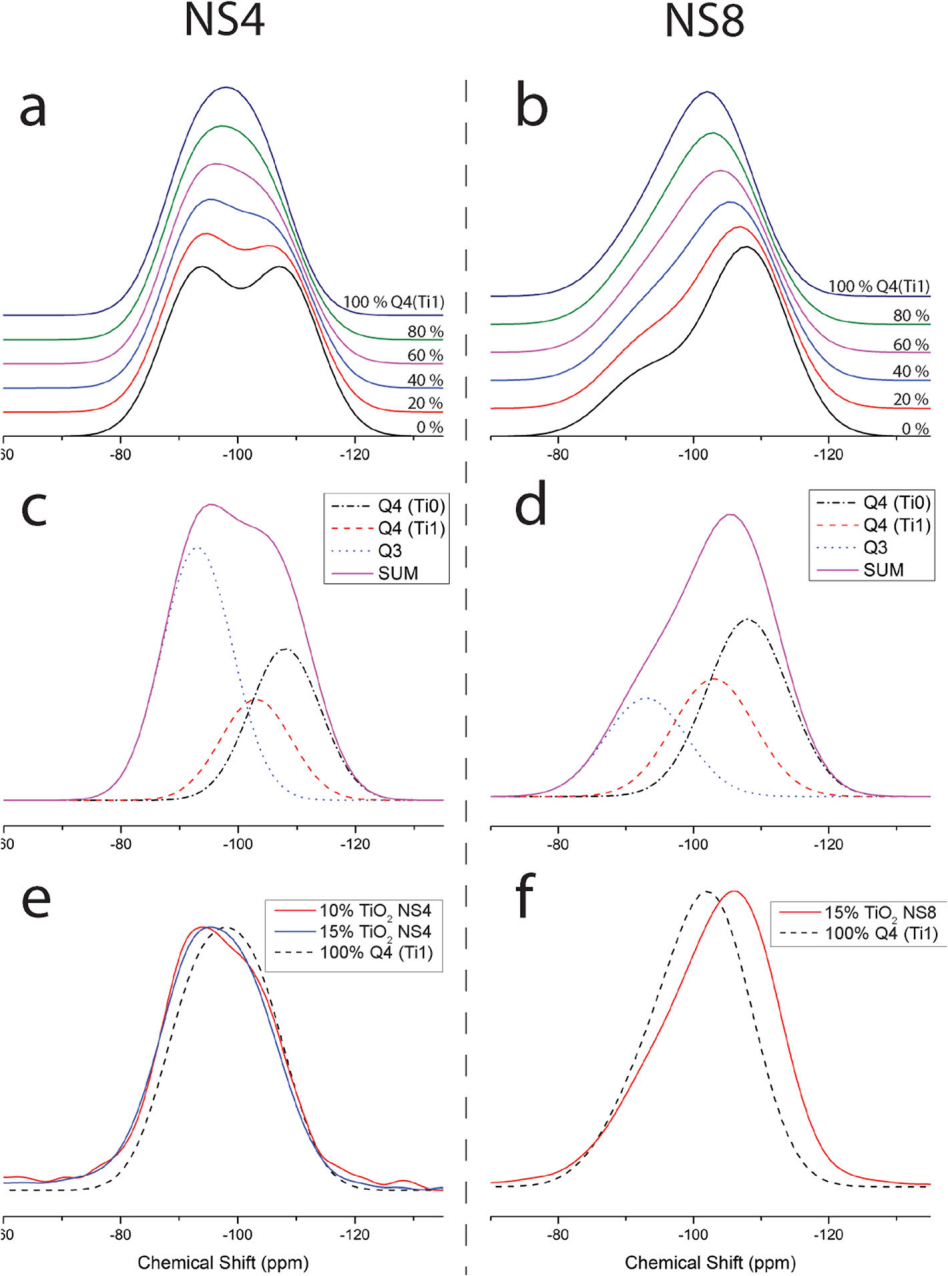
Simulated ^29^Si NMR spectra for Ti-bearing Na-silicate glasses, generated using Gaussian peaks at the positions and shapes of the Ti-free **a** NS4 and **b** NS8 glasses, by varying the proportions of Q^4^ (Ti = 1) and Q^4^ (Ti = 0) peaks. The peaks that compose these model spectra at 40% Q^4^ (Ti = 1) and 60% Q^4^ (Ti = 0) are shown in 8 **c** and 8 **d**. Increasing Ti content in the simulations is represented by the growth of a third Q^4^ peak at a higher frequency as a result of nearest-neighbor interactions with ^[4]^Ti nearest neighbors. The simulations are similar to the actual spectra, with the notable difference that the simulations over-estimate the amount of Q^4^ Si with ^[4]^Ti nearest neighbors Q^4^ (Ti = 1). **e**, **f** If Ti was incorporated entirely by the Q^4^ (Ti = 1) mechanism, then observed spectra would be reflective of 100% Q^4^ (Ti = 1) at high Ti content in NS4 and NS8 glasses. However, NS8 glass with 15 mol% TiO2 and NS4 glass with 10 and 15% TiO2 which would have all Si in Q^4^ (Ti = 1) at these concentrations are still significantly far from Q^4^ (Ti = 1). This over-estimation suggests that Ti is clustering at relatively low bulk TiO_2_, an observation that is supported by the Ti XAFS observations of significant ^[5]^Ti in the NS8 glasses

In Fig. 8a and b, model spectra of NS4 and NS8 glasses are presented using identical Gaussian peak shapes and fixed frequencies for the Q^3^ (Ti = 0), Q^4^ (Ti = 0), and Q^4^ (Ti = 1) peaks. Figure 8c and d shows a spectral deconvolution of these peaks at 40% (by area) Q^4^ (Ti = 1) and 60% Q^4^ (Ti = 0). With titanium addition, we assume that silicon Q^4^ species are systematically transformed from having 0 neighboring “Ti^4+^” (Q^4^ Ti = 0) up to 100% Q^4^ with one neighboring “Ti^4+^” (Q^4^ Ti = 1). Note that for NS4 with 100% Q^4^ (Ti = 1), the corresponding total titanium concentration would be 6.25 mol% TiO_2_ (e.g., 1 Ti for every 6 Q^4^ Si), for the more silica rich (and Q^4^ rich) NS8, the corresponding total concentration of titanium at 100% Q^4^ (Ti = 1) would be 9.5 mol% TiO_2_. For titanium concentrations above these values, either Q^4^ (Ti = 2) species would have to form which by comparison with Q^4^ (Al = 2) (Fig. 2) would lead to an apparent increase in intensity around Q^3^ (Ti = 0), or Q^3^ (Ti = 1) would have to form which by comparison with the effects of aluminum would lead to the prediction of increased intensity at ~− 80 ppm. If TiO_2_ begins to cluster into titanium oxide rich domains, the presence of such would not be predicted to influence or perturb the ^29^Si NMR spectra.

The forward model of the NS4-TiO_2_ system (Fig. 8a) is similar to the measured ^29^Si NMR data (Fig. 3) at low Q^4^ (Ti = 1) concentrations where a progressive shift of Q^4^ to slightly higher frequency occurs with titanium addition. Interestingly, the actual ^29^Si spectra for NS4 with 15% Ti (Fig. 3) looks most similar to model at 60–70% Q^4^ (Ti = 1) (Fig. 8a). This suggests Si–Ti interactions saturate with Q^4^ (Ti = 1) at ~ 4–5 mol% Ti, suggesting that the remaining (~ 10 mol% at 15 mol% TiO_2_ oxide) Ti^4+^ forms oxide clusters whose size and number increase up to the maximum titanium concentration without further perturbation to the ^29^Si spin system.

The forward model of the NS8-TiO_2_ system (Fig. 8b) is also similar to the measured ^29^Si NMR data (Fig. 4) at low Q^4^ (Ti = 1) concentrations. At 100% Q^4^ (Ti = 1), the apparent shift in “Q^4^” exceeds what is observed in the actual NS8 ^29^Si NMR spectra (Fig. 4). At 50% Q^4^ (Ti = 1), one observes a model spectrum that is very similar to that of NS8 with 15 mol% TiO_2_ (Fig. 8b). As is the case with the NS4-TiO_2_ system, this suggests that Si–Ti interactions saturate with Q^4^ (Ti = 1) again at 4–5 mol% TiO_2_. Again, at 15 mol% TiO_2_, the remaining ~ 10 mol% TiO2 must reside in Ti oxide clusters whose presence would not be evident in the ^29^Si NMR spectra (Figs. 3 and 4).

The analyses of the forward models in comparison with the actual ^29^Si NMR spectra (Fig. 8e, f) indicate that the perturbation to silicate is much less than what is possible given the amount of TiO_2_ available. In both the case of NS4 and NS8 TiO_2_ series, the principle perturbation due to TiO_2_ addition is the formation of a modest amount of Q^4^ (Ti = 1) groups. It is noted that the Pauling radii of Si^4+^ is 41 pm, Al^3+^ is 50 pm, and Ti^4+^ is 68 pm (Pauling 2014). The much larger radius of Ti^4+^ relative to Si^4+^ may limit titanium substitution to Si^4+^ tetrahedral to no more than one Ti^4+^ [e.g., Q^4^ (Ti = 1)]. The ^29^Si NMR spectra indicate that titanium oxide also likely begins to cluster with itself even at relatively low bulk titanium concentrations (Farges 1999; Kim et al. 2000). Obviously, the ^29^Si NMR spectra only report on what directly perturbs silicon, so as titanium oxide begins to cluster with titanium oxide; such clustering will “dilute” the perturbation of the silicate component of the melt/glass and will minimize what is observed with ^29^Si solid state NMR. Large Ti clusters would likely result in the formation of higher order Ti coordination (octahedral Ti) that is not observed, suggesting the clustering occurs in small localized regions dispersed throughout the glass matrix. However, these regions also must be large enough that no significant Q^4^ (Ti = 2) or Q^4^ (Ti = 3) peaks are generated at high bulk TiO_2_ content as a result of Ti dispersal between SiO_2_ tetrahedra. The fact that there is no evidence for Q^3^ (Ti = 1) in the ^29^Si NMR spectra suggests that the Q^4^ (Ti = 1) species may signify the interface between alkali silicate oxide phases and titanium oxide clusters.

## Conclusions, implications, and importance for natural systems

This work highlights the complexity of Ti incorporation in silicate glasses, and the variable mechanisms by which Ti can be incorporated into melts. In natural magmatic liquids, changing solubility mechanisms could significantly influence the stability of Ti-rich phases and the calculation of Ti^4+^ activity in rutile-undersaturated melts (Ghiorso and Gualda 2013). A better understanding of how Ti^4+^ is incorporated into glasses can also help explain the physical phenomena (e.g., compressibility, tensile strength) that occur as Ti^4+^ is incorporated into glass compositions (Scannell et al. 2016).

Although the observations presented here are not directly applicable to natural silicate glasses (e.g., rhyolites, basalts), they provide insight into important processes operating in natural systems. In particular, the observations shown here clearly demonstrate a change in the solubility mechanism for Ti^4+^ in glasses with changes in the bulk TiO_2_ content of the glass. Thermodynamically, changes in the solubility mechanism of Ti^4+^ in glasses will change the activity coefficient for the TiO_2_ fusion reaction as a function of TiO_2_ content. In turn, this will play a significant role in the calculation of Ti^4+^ activity in silicate melts. Because accurate estimation of Ti^4+^ activity is required for numerous applications (phase stability, trace-element thermobarometry), it is important for similar observations to be made on natural systems to gain a more complete picture of the solubility mechanisms for Ti^4+^ in melts and glasses. Specifically, changes in solubility mechanisms could result in the overestimation of TiO_2_ activity in rutile-undersaturated melts using rutile-saturation modeling (Ackerson and Mysen in press). Employing NMR in combination with Ti *K*-edge XAFS for other model systems could provide specific evidence to help explain the anomalous behavior of Ti^4+^ in glasses.

The applicability of the combined use of these two techniques will be diminished in systems (e.g., rhyolites), where the Ti saturation concentration at magmatic temperatures is too low to exert meaningful or detectible influence on ^29^Si NMR spectra. However, these combined techniques could be useful in understanding how Ti^4+^ is incorporated into more mafic melts, where Ti^4+^ saturation concentrations are significantly higher (Gaetani et al. 2008). Utilizing these techniques could shed light on rutile solubility in mafic systems, inform our understanding of the processes leading to Nb-Ta anomalies, and bolster our understanding of the origins of high-Ti lunar glasses (Van Orman and Grove 2000).

## Supplementary information

**Additional file 1: Supplementary Figure S1.** Ti XANES spectrum of an NS8 glass with 15 mol. % TiO_2_, compared with a spectrum from ^[6]^Ti-bearing crystalline titanite (CaTiSiO_5_). The sharp white line peak in the titanite spectrum above the absorption edge is an indicator of the crystalline nature of the titanite. The broad edge feature in the Ti15NS8 spectrum suggests the material is amorphous. **Supplementary Figure S2.** Ti XANES spectrum of NS8 glasses with 10 mol % TiO2 synthesized from 1186, 1250, and 1580 °C. All glasses produced near-identical spectra, suggesting that there is no effect of dwell temperature on the solubility mechanism of Ti in NS8 glasses.

## Data Availability

The dataset supporting the conclusions of this article is included within the article (and its additional files).
