# Supplementary material for: 29Si solid state NMR and Ti K-edge XAFS pre-edge spectroscopy reveal complex behavior of Ti in silicate melts
Source: Prog Earth Planet Sci. 2020 Mar 18;7(1):14. doi: 10.1186/s40645-020-00326-2 (PMC7324145; doi:10.1186/s40645-020-00326-2)
Supplement: Supplementary file 1 — Additional file 1: Supplementary Figure S1. Ti XANES spectrum of an NS8 glass with 15 mol. % TiO2, compared with a spectrum from [6]Ti-bearing crystalline titanite (CaTiSiO5). The sharp white line peak in the titanite spectrum above the absorption edge is an indicator of the crystalline nature of the titanite. The broad edge feature in the Ti15NS8 spectrum suggests the material is amorphous. Supplementary Figure S2. Ti XANES spectrum of NS8 glasses with 10 mol % TiO2 synthesized from 1186, 1250, and 1580 °C. All glasses produced near-identical spectra, suggesting that there is no effect of dwell temperature on the solubility mechanism of Ti in NS8 glasses. [file 40645_2020_326_MOESM1_ESM.docx]

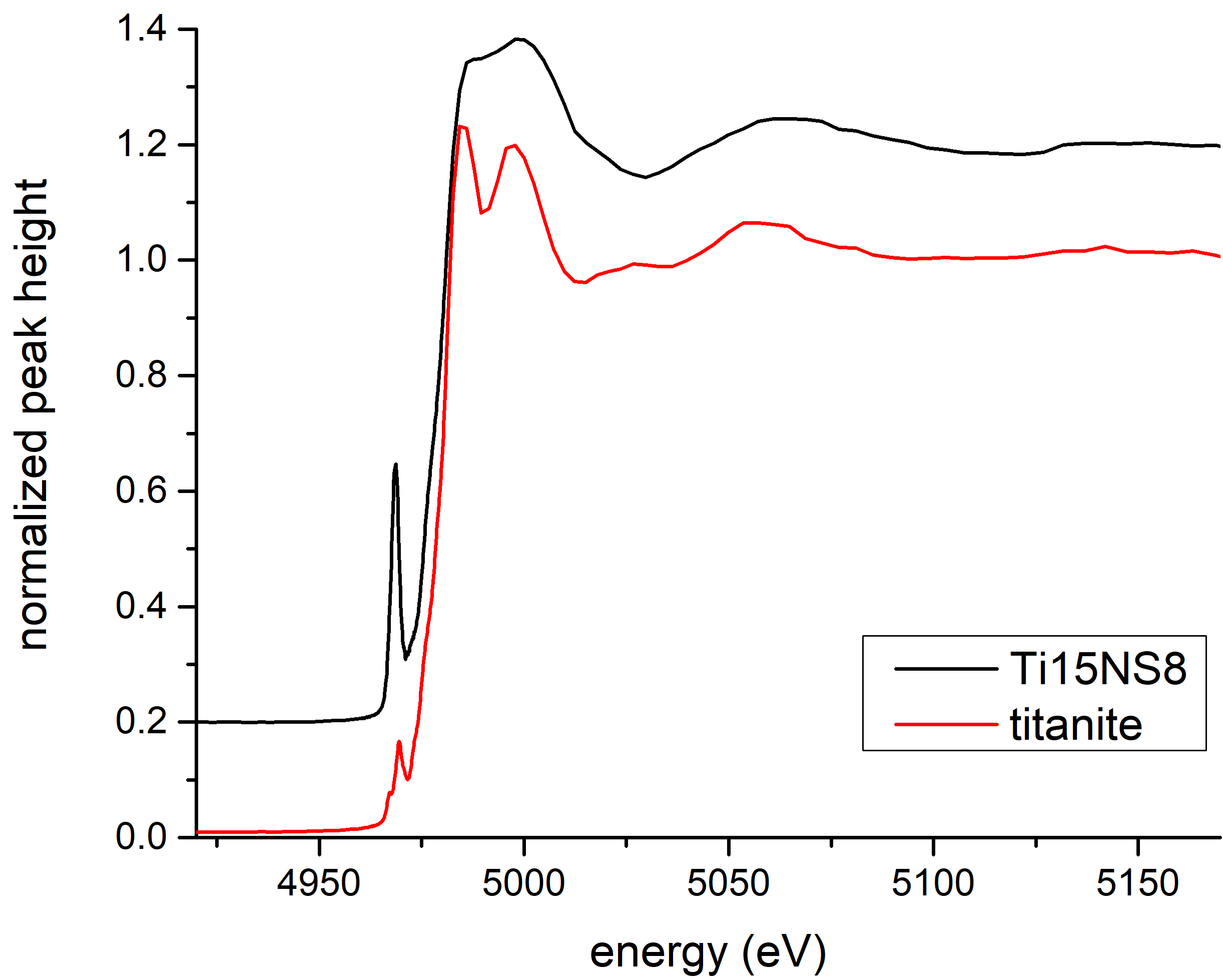


**Supplementary Figure S1**: Ti XANES spectrum of an NS8 glass with 15 mol. % TiO_2_, compared with a spectrum from ^[6]^Ti-bearing crystalline titanite (CaTiSiO_5_). The sharp white line peak in the titanite spectrum above the absorption edge is an indicator of the crystalline nature of the titanite. The broad edge feature in the Ti15NS8 spectrum suggests the material is amorphous.


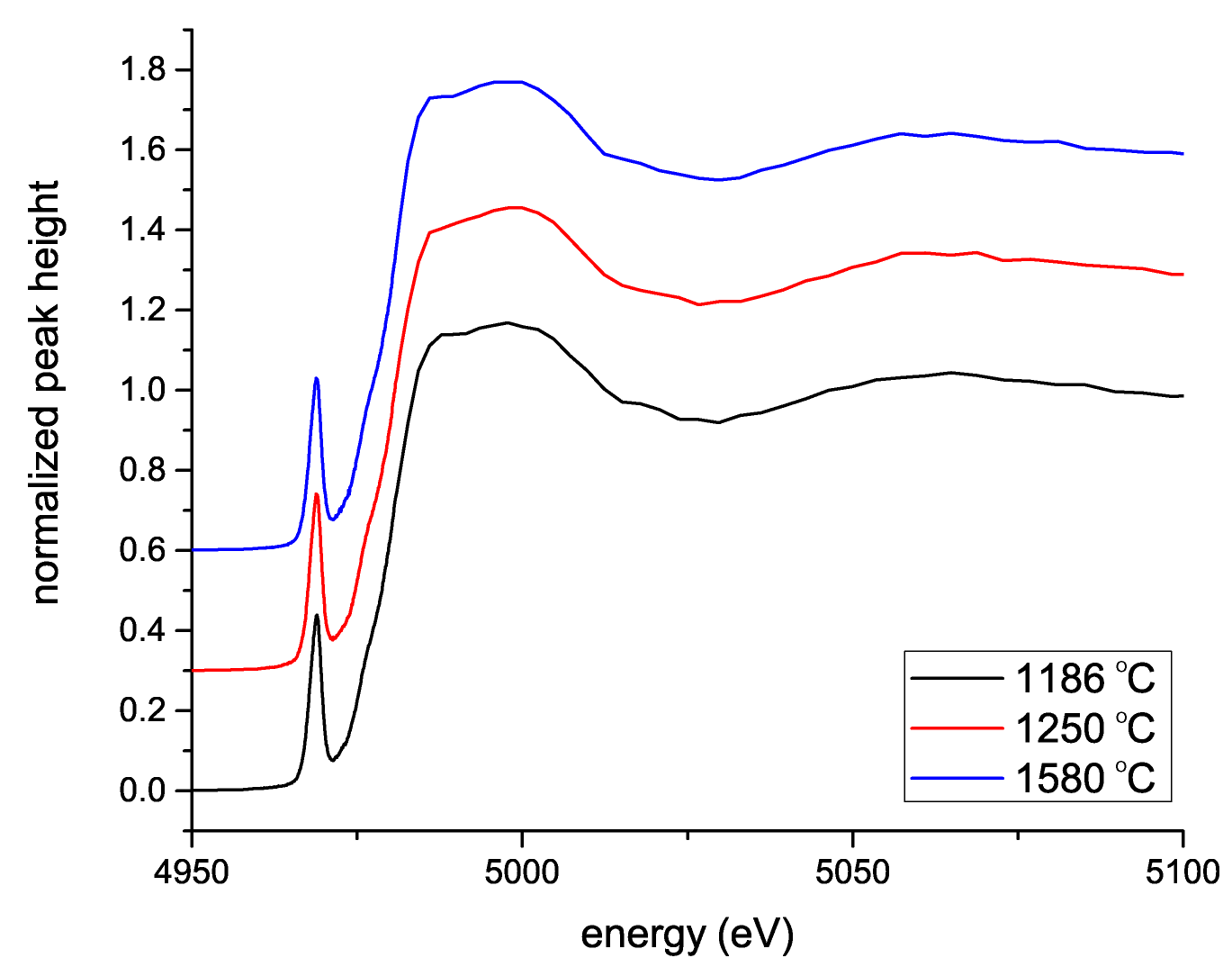


**Supplementary Figure S2**: Ti XANES spectrum of NS8 glasses with 10 mol % TiO2 synthesized from 1186, 1250, and 1580 °C. All glasses produced near-identical spectra, suggesting that there is no effect of dwell temperature on the solubility mechanism of Ti in NS8 glasses.
